# Supplementary material for: Epithelial changes of congenital intestinal obstruction in a rat model
Source: PLoS One. 2020 Apr 30;15(4):e0232023. doi: 10.1371/journal.pone.0232023 (PMC7192479; doi:10.1371/journal.pone.0232023)
Supplement: Attachment — Submitted filename: Response to reviewers.doc [file pone.0232023.s003.doc]

Limoges, February 14 2020

To the Editorial Board of *PLOS ONE*,

We thank the reviewers and the editors for pointing out that our manuscript would benefit considerably from a number changes. We have, therefore, carefully revised our manuscript in accordance with the reviewers’ comments. Our point-by-point responses to the reviewers’ concerns are provided in the following pages and highlighted in the revised manuscript.

We hope that in light of these changes that the manuscript is now suitable for publication in *PLOS ONE*.

We look forward to hearing from you soon.

Sincerely yours,

Quentin Ballouhey on behalf of the authors.

Service de chirurgie viscérale pédiatrique, Hôpital des Enfants,

8 avenue Dominique Larrey 87042

Limoges Cedex, France

[q.ballouhey@gmail.com](mailto:q.ballouhey@gmail.com)

**Response to Reviewer 1**

**Reviewer 1: Manuscript appears to be well explained and contains all the statistically significant supporting data. It would be good to mention the number of animals used for each group, while explaining materials method (though it has been mentioned later in results). Please, carefully read the manuscript and prefer to avoid using repetitive words (like "impaired" used twice in a single sentence). Also avoid repetitive use of "the" at many places (like "The ratio of the target genes to the reference genes"). Rest seems to be fine.**

We thank Reviewer 1 for their very useful comments. As suggested in the comments, we have changed the following:

-“Impaired” has been replaced with “Altered” in the introduction section

-The sentence “The ratio of the target genes to the reference genes was calculated and expressed as 2^-ΔCt^.” Was modified to “Target gene/reference gene ratios were calculated and expressed as 2^-ΔCt^.”

-The style of the entire manuscript style has also been checked.

**Response to Reviewer 2**

**Reviewer 2: The manuscript entitled “Epithelial changes of congenital intestinal obstruction in a rat model” by Ballouhey et al. has described the effect of surgically induced atresia in rat embryos. Different models of atresia have been reported, however, most of them focused on morphological changes. The authors tried to advance previous reports on morphological changes by taking a gene expression profile-based approach together with morphological examinations. However, in my opinion, the manuscript fails to address the specific scientific hypothesis. The conclusions, and the title of the manuscript are not supported by data.**

**Response to Reviewer 2**

**1. Overall representation of the manuscripts looks more incoherent. The result sections are very confusing and redundant.**

Author’s response: We apologize for the fact that Reviewer 2 found the Results section to be confusing. In light of this remark, we have changed the followings points in the Results paragraph:

-“Analysis of global gene expression” section has been moved in the first part of the paragraph.

-The presentation of the table illustrating the morphometric results has been modified.

-Throughout the entire paragraph, “Microvilli” has been substituted with “Villi” as Microvilli can only been detected by electron microscopy.

-The “Enterocyte cell brush border” section has been modified and the last sentence has been deleted “Sucrase isomaltase…(S1 Table)”.

**2. Sample size for most of the data are very small (n=3). Previous studies by Fourcade et al. had used n > 10 in the rat model.**

Author’s response: Indeed, the sample size was n = 3 or = 4, as the guidelines for animal experimentation stipulate that as few animals as possible are used. The aim of the experiment was to use a variety of techniques rather than a large number of animals. The transcriptomic experiment required n = 3 for each group, which meant using more than 18 operated animals, not including the high mortality rate during the surgical procedures. In previous published studies, the number of animals was higher but only a single technique was used, for example Fourcade et al. only performed an optical microscopy analysis.

**3. Table 2: The statistical power of this data is not so convincing considering the small sample size (n=3). It is difficult to assume the gaussian distribution of the data with the small sample size and with high variation. Therefore, I suggest non parametric test for this set of data.**

Author’s response: We fully agree with this remark. Consequently, we have opted to use a non-parametric test and we have made the following changes:

-The sentence corresponding to the statistical analysis has been changed in the Methods section: Differences in the area between the proximal and the distal segment have been analyzed quantitatively using the Wilcoxon test.

-The data in the Results section corresponding to Table 3 have been changed.

A qualified statistician has now reviewed the statistics of the manuscript and this individual has hence been added to the list of authors.

**4. Table 3: number of deferentially expressed gene does not make any scene unless they suggest any biological function or pathway. I suggest a gene ontology analysis of the deferentially expressed gene to better represents the biological changes.**

Author’s response: the objective was not to produce biologically significant results but to determine which segment was the most impaired. In accordance with the reviewer’s comment, we have made the following modification:

-The beginning of the Introduction section has changed to “The aim of our study was to determine whether the proximal or the distal segment was the most impaired and …to analyze…”

-The Results section has also been modified and the “Analysis of global gene expression” section along with the related Table (Table 3 becoming Table 2) has been moved in the first part of this paragraph.

**5. Is the gene expression data deposited in any data bank?**

Author’s response: Yes, the data are available online. The microarray analysis of this study was performed at the genomic platform (GENOMIC’S) of the Institut Cochin in Paris (22 Rue Mechain, 75014 Paris) and it has been assigned accession number E-MTAB-5981 in ArrayExpress.

**6. Figures should be more organized and properly labelled for the clarity of the readers.**

Author’s response: We apologize for the fact that Reviewer 2 found our pictures to be in want of improvement. In accordance to this, all of the patterning schematics of the figures have been changed to improve their appearance: the labelling of the Figures 1 and 2 has been modified. A legend has been added for each picture and the scale bars have been modified.

**7. Minor concerns. Please provide HPRT and TBP primer sequence in table 1.**

Author’s response: the HPRT and TBP primer sequences are now provided in Table 1.
