# Supplementary material for: Epithelial changes of congenital intestinal obstruction in a rat model
Source: PLoS One. 2020 Apr 30;15(4):e0232023. doi: 10.1371/journal.pone.0232023 (PMC7192479; doi:10.1371/journal.pone.0232023)
Supplement: Attachment — Submitted filename: Response to reviewers.doc [file pone.0232023.s004.doc]

Limoges, April 6 2020

To the Editorial Board of *PLOS ONE*,

We thank the reviewers and the editors for pointing out that our manuscript would benefit considerably from a number changes. We have, therefore, carefully revised our manuscript in accordance with the reviewers’ comments. Our point-by-point responses to the reviewers’ concerns are provided in the following pages and highlighted in the revised manuscript.

We hope that in light of these changes that the manuscript is now suitable for publication in *PLOS ONE*.

We look forward to hearing from you soon.

Sincerely yours,

Quentin Ballouhey on behalf of the authors.

Service de chirurgie viscérale pédiatrique, Hôpital des Enfants,

8 avenue Dominique Larrey 87042

Limoges Cedex, France

[q.ballouhey@gmail.com](mailto:q.ballouhey@gmail.com)

**Response to Reviewer 2**

**Reviewer 2: Manuscript appears to be well explained and contains all the The authors have significantly improved the revised manuscript. I would suggest that the following analysis to be included in the manuscript for better elucidation of the intestinal differentiation in control embryos. As the authors have performed microarray with different stages of control samples, namely ED15, ED17, ED19 and E21, it is important to show differential gene expression of all three stages (i.e. ED17,19, 21) compare to ED15 and associated pathways instead of just number of genes in ED21 vs ED15 (table 2).**

Author’s response:

We thank Reviewer 2 for their very useful comments.

We fully agree with this remark.

Consequently, we have implemented the intermediate results of microarray E15-17-E19-E21 for kinetic controls in Table 2.

We have implemented the intermediate results of microarray E15-17-E19-E21 kinetic controls for specific pathways in Table S1.
